# Long‐Term Societal Costs After Births Before 24 Weeks of Gestation in Sweden

**DOI:** 10.1111/apa.70527

**Published:** 2026-03-31

**Authors:** Chatarina Löfqvist, Boubou Hallberg, Ulrika Sjöbom, David Ley, Liv Vallin, Eva Morsing, Karin Sävman, Ann Hellström, Hanna Gyllensten

**Affiliations:** ^1^ Institute of Health and Care Sciences, Sahlgrenska Academy University of Gothenburg Gothenburg Sweden; ^2^ University of Gothenburg Centre for Person‐Centred Care ‐ GPCC University of Gothenburg Gothenburg Sweden; ^3^ Department of Clinical Neuroscience, Institute of Neuroscience and Physiology, Sahlgrenska Academy University of Gothenburg Gothenburg Sweden; ^4^ Sahlgrenska University Hospital Gothenburg Sweden; ^5^ Department of Pediatrics, Institute of Clinical Sciences Skåne University Hospital Lund Lund Sweden; ^6^ Department of Neonatology, the Queen Silvia Children's Hospital Sahlgrenska University Hospital Gothenburg Sweden; ^7^ Department of Pediatrics, Institute of Clinical Sciences, Sahlgrenska Academy University of Gothenburg Gothenburg Sweden; ^8^ Department of Ophthalmology Sahlgrenska University Hospital Gothenburg Sweden

**Keywords:** birth before 24 weeks, extremely preterm birth, health economics, societal costs

## Abstract

**Aim:**

Extremely preterm birth is associated with high morbidity and long‐term support needs. This study estimated long‐term medical and non‐medical costs throughout childhood among children born before 24 weeks of gestation in Sweden.

**Methods:**

This nationwide register‐based study included 344 infants born before 24 weeks of gestation in Sweden between 2007 and 2018 who survived at least one year. Individual‐level data from national health registers and social insurance records were linked to estimate costs of hospital care, outpatient care, prescribed drugs and parental support until the end of 2022. Costs were analysed from a societal perspective and expressed in 2022 Swedish kronor.

**Results:**

The mean follow up time was 7.8 years. Average total costs per child were 1.68 million Swedish kronor in the first year of life, with medical care accounting for more than 80% of expenditures. Non‐medical costs increased during childhood and exceeded medical costs from approximately four years of age. Neonatal morbidities, including bronchopulmonary dysplasia, treated retinopathy of prematurity, and severe intraventricular haemorrhage, were associated with sustained long‐term costs.

**Conclusion:**

Birth before 24 weeks of gestation was linked to high long‐term costs. Early costs were related to hospital care, while long‐term costs increasingly reflected social and welfare support.

## Summary

- Birth before 24 weeks of gestation is associated with major health complications and long‐term support needs, but the societal costs of these births are poorly described.
- In this nationwide Swedish study, medical costs were highest in the first year of life, while non‐medical costs related to family support and social insurance increased during childhood and exceeded medical costs after approximately four years.
- Morbidities were important indicators of long‐term costs.

## 1 Introduction

Birth before 24 weeks of gestation is associated with high risks of mortality and long‐term morbidity, including respiratory, neurological and sensory complications [1, 2, 3]. Advances in perinatal and neonatal care have improved survival. In Sweden, active resuscitation policies at 22 weeks of gestation have been implemented [4]. However, these improvements in survival also bring challenges for families, healthcare systems and society.

Children born at the threshold of viability often experience prolonged hospitalisations and require specialised follow up. Studies from Canada, the United States and the United Kingdom reported high costs during infancy and sustained non‐medical costs into later childhood and adolescence [5, 6, 7, 8]. Data from the United States showed that prematurity‐related morbidities were major drivers of inpatient costs. Swedish cohort studies reported differences in survival between infants born at 22 and 23 weeks of gestation [9], while other studies showed that neonatal morbidity remained common among extremely preterm infants [3]. This highlights the importance of gestational age‐specific analyses when estimating long‐term societal costs. However, evidence on the long‐term economic impact in Sweden remains limited, underscoring the need for register‐based studies in contemporary Swedish cohorts. The country's national health and social registers allow comprehensive tracking of both medical and non‐medical costs at the individual level. Nevertheless, there is currently no published cost‐of‐illness analysis specifically focused on children born before 24 weeks of gestation. Prior studies often grouped infants born at the lowest gestational ages together with those born slightly later. This reduced the representation of extremely immature infants and may have underestimated their substantially higher costs. Swedish population‐based studies [10] and the Finnish PERFECT project [11] provided important insights into survival, morbidity patterns and early healthcare use among very preterm infants. However, neither the Swedish nor the Finnish cost analyses included enough infants born before 24 weeks to allow detailed, gestational age‐specific cost estimates for this group.

The aim of this study was to estimate the medical and non‐medical costs of illness throughout childhood among children born before 24 weeks of gestation in Sweden.

## 2 Patient and Methods

The cohort study includes all infants born before 24 weeks of gestation in Sweden between 2007 and 2018 who survived at least one year and uses retrospective register data linked to data extracted from medical records to estimate the medical and non‐medical costs of illness from a societal payer perspective. The manuscript adheres to the Consolidated Health Economic Evaluation Reporting Standards [12].

### 2.1 Data Sources and Linkage

Sweden's universal healthcare system is funded primarily through taxes [13]. For infants and children, healthcare services and prescribed medications are provided free of charge to families; reimbursed by the regions. This includes neonatal intensive care at tertiary centres during infancy, follow up at regional hospitals and care delivered in both public and most private outpatient settings [14]. In addition, the Swedish Social Insurance Agency compensates parents for income loss and additional expenses associated with their child's medical condition, based on structured needs assessments [15].

Infants were identified via the Swedish National Registry for Retinopathy of Prematurity [16], which captures more than 98% of infants undergoing retinopathy of prematurity screening in Sweden. Individual‐level data were then linked across several national and regional registers using the unique personal identity number assigned to all residents, with linkage performed by the National Board of Health and Welfare [17]. Pseudonymised data were made available to the research team, while the National Board of Health and Welfare board retained the encryption key.

Linked registers included the Swedish Medical Birth Register, which provided perinatal and delivery characteristics [18], the National Patient Register for inpatient and physician‐led outpatient care [19], the Prescribed Drug Register for dispensed medications and associated costs [20], and the Cause of Death Register to identify mortality when applicable [21]. All registers were held by the National Board of Health and Welfare.

Additional data on primary care and non‐physician‐led outpatient visits were available for a regional subset of the cohort (*n* = 78) through the Västra Götaland VEGA healthcare database [22].

This subgroup was used for a sensitivity analysis to explore healthcare contacts not fully captured in the National Patient Register and was not extrapolated to the full study population. Records from the Swedish Social Insurance Agency were used to capture parental benefits, care allowances and other income‐related transfers.

### 2.2 Cost Analysis

All costs were estimated in 2022 value Swedish Krona. Medical costs encompass healthcare and prescription drug expenses. Inpatient and specialised outpatient care costs were calculated from national diagnosis‐related group codes in the National Patient Register and transformed using diagnosis‐related group weights with the average cost per 1.0 diagnosis‐related group unit from Swedish statistics 68 442 Swedish Krona [23]. Such costs are assigned to each healthcare encounter based mainly on recorded diagnoses and procedures during each admission or visit and will therefore vary between individuals. We applied the national diagnosis‐related group weights for each code and did not perform additional outlier trimming beyond what is inherent in the Swedish diagnosis‐related group reimbursement system. A primary care physician visit in 2022 cost 2442 Swedish Krona. Visits to other healthcare professionals were weighted as 40% of the cost of a physician visit, and indirect costs were weighted as one‐third of the cost of a physical visit to the corresponding professional [24]. Dispensed drug costs were estimated from the total cost of drugs dispensed in community pharmacies both reimbursed costs and out‐of‐pocket payments, corresponding to 2% of the costs. Non‐medical costs included transfer payments intended to reimburse expenses related to adjustments of home, car or other costs for the families, attendance allowance when needing assistance services and lost income due to caring for the sick child. Transfer costs were inflated to 2022 value using consumer price indices.

### 2.3 Statistical Analysis

Analyses were conducted using Stata 18.5 (StataCorp LLC, USA); illustrations were created using R‐Statistics Software version 4.3.0 (R Foundation, Vienna, Austria), with the ggplot package. Descriptive statistics were used to summarise population characteristics, as well as costs by components and age. Cost components were summarised and reported by age as means and standard deviations or as accumulated costs. Costs were examined across selected subgroups to explore variation in cost patterns. Subgroups were defined by gestational age at birth, comparing infants born at 21–22 weeks with those born at 23 weeks, by sex, by mode of birth and by major neonatal morbidities including bronchopulmonary dysplasia, treated retinopathy of prematurity and intraventricular haemorrhage grade 3–4. No formal statistical hypothesis testing was performed. The subgroup analyses are descriptive and intended to explore cost patterns rather than to assess statistical significance.

Ethical approval for the data collection and analysis was obtained under protocol numbers (EPM‐Dnr. 2020–00231, Dnr. 2020–02252, Dnr. 2021–06161‐02). PPI: Patients and the public were not directly involved in this register‐based study, which aims to provide a basis for future research initiatives in collaboration with relevant interest holders.

## 3 Results

### 3.1 Population Characteristics

**FIGURE 1:**
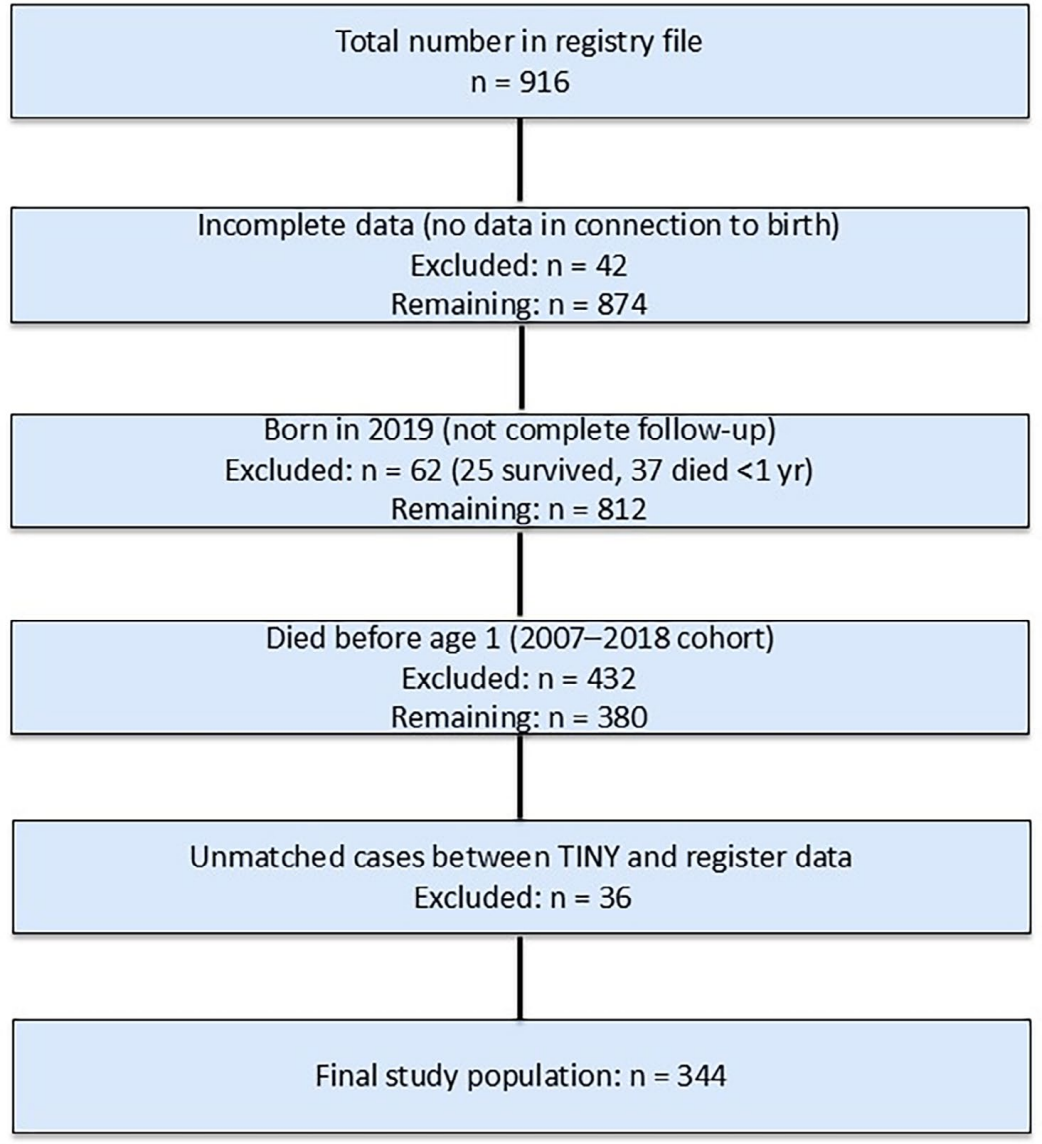
Flowchart.

The study population consisted of 344 children born before 24 weeks of gestation who survived for at least one year (Figure 1). Of these, 52.6% were boys (Table 1). The mean gestational age was 23.3 (95% Confidence interval (CI) 23.2–23.3 weeks), and the average birth weight was 567 g (95% CI 559.1 g–575.4 g). Severe neonatal morbidities were common: 85.1% of the children had bronchopulmonary dysplasia, 42.4% had received treatment for retinopathy of prematurity and 16.9% had intraventricular haemorrhage grade 3–4. The oldest child with complete follow‐up data from 2007–2022 was 14 years of age and the mean follow up time was 7.8 years (95% CI interval 7.5–8.2 years).

**TABLE 1:** Demographics of the study population (*n* = 344).

Variable	Value	Percentage / 95% CI
Total infants surviving > 1 year	344	
Mean gestational age at birth (weeks)	23.3	95% CI 23.2–23.3
Mean birth weight (grams)	567	95% CI 559.1–575.4
Girls	163	47.4%
Boys	181	52.6%
Twins	51	14.8%
Caesarean section	81	23.6%
Treated for ROP	146	42.4%
BPD diagnosis	279	85.1%
IVH grade 3–4	58	16.9%
NEC surgery	47	13.7%
PDA surgery	168	50.0%
PDA medically treated	100	56.8%
Mean follow‐up time (years)	7.8	95% CI: 7.5–8.2

Abbreviations: BPD, Bronchopulmonary dysplasia; IVH, Intraventricular haemorrhage; NEC, Necrotising enterocolitis; PDA, Patent ductus arteriosus; ROP, Retinopathy of prematurity.

### 3.2 Costs of Illness

**FIGURE 2:**
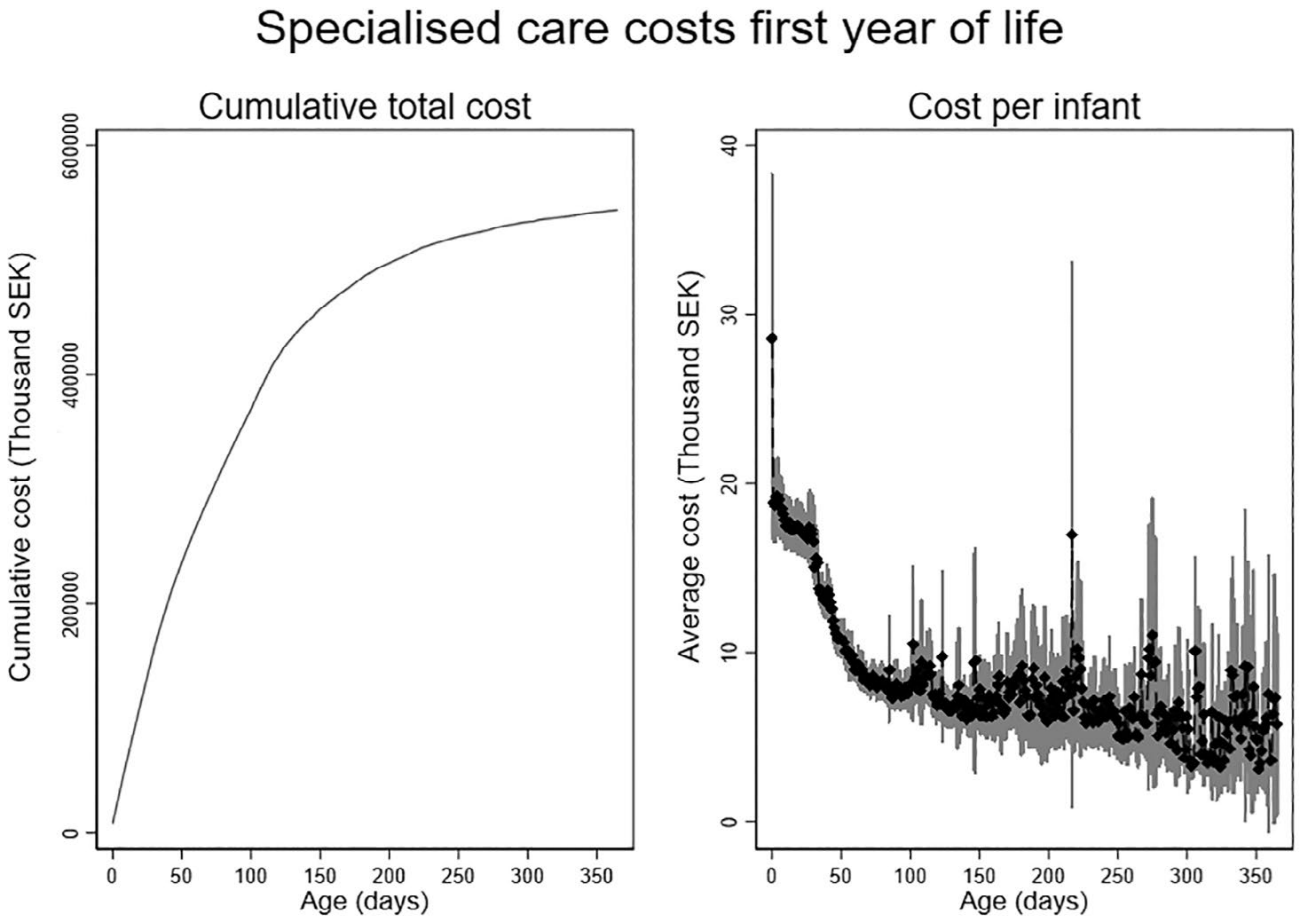
Total (left) and average cost per day (right) for specialised care during infancy, among children born at < 24 weeks gestational age. SEK Swedish Krona.

**FIGURE 3:**
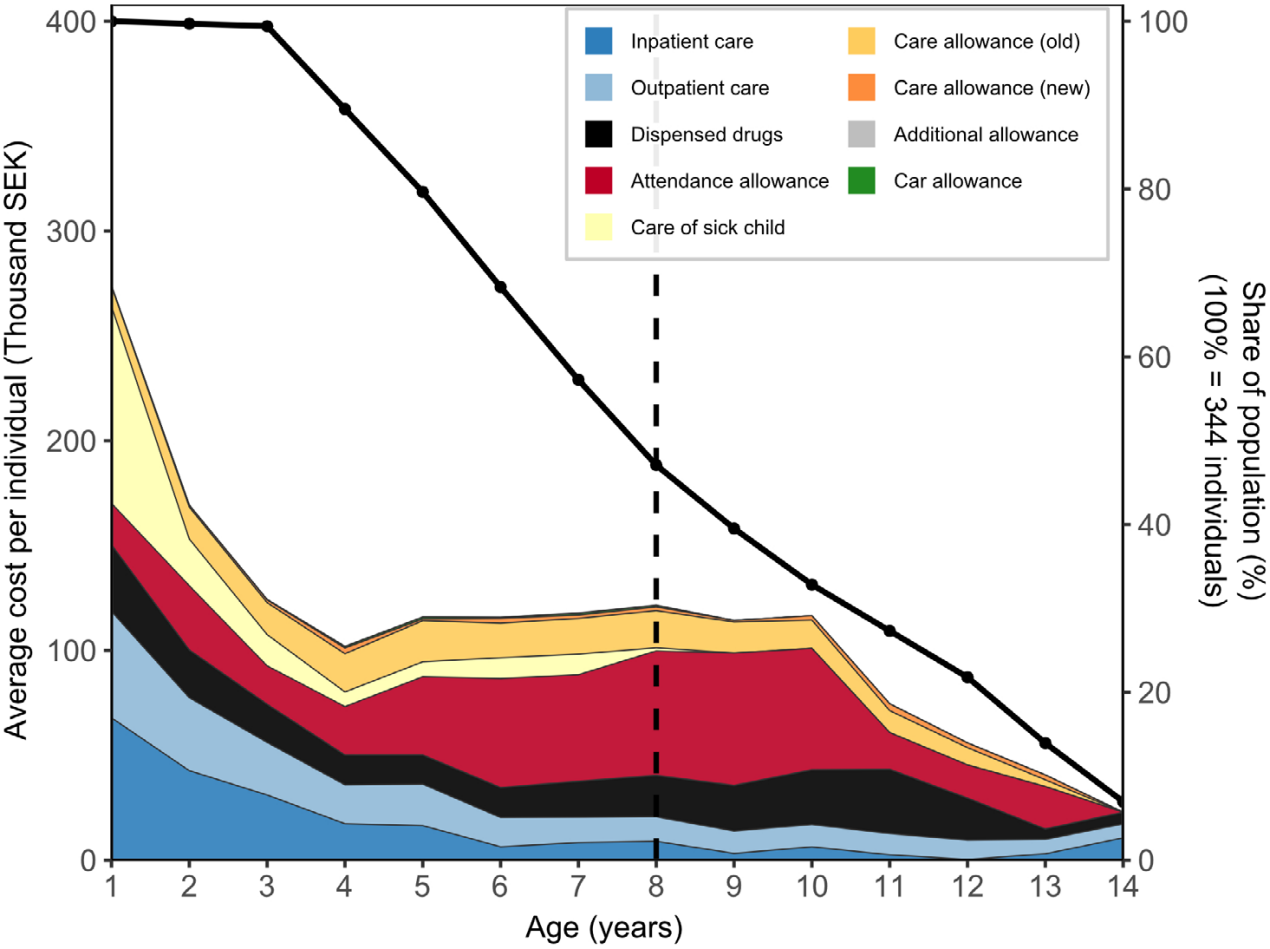
Mean cost by age after infancy, among children born < 24 weeks gestational age. The black dotted line indicates the percentage share of the total population. SEK Swedish Krona.

Medical costs were highest during infancy and early childhood, with more than 80% of healthcare expenditure occurring during the first year of life. Detailed counts of healthcare contacts and social insurance benefits by age are provided in Table S1. As illustrated in Figure 2, specialised inpatient care dominated costs during infancy. Over time, non‐medical costs increased and exceeded medical costs from approximately four years of age (Table 2). During the same period, inpatient care costs declined, while outpatient care and social support‐related costs became more prominent (Figure 3).

**TABLE 2:** Mean medical and non‐medical costs by age.

Year of age	Numbers	Medical cost TSEK mean (95% CI)	Non‐medical cost TSEK mean (95% CI)	Total cost TSEK mean (95% CI)
0	344	1607 (1513–1701)	69 (63–75)	1676 (1581–1771)
1	344	150 (126–175)	123 (102–145)	274 (239–308)
2	343	100 (80–121)	69 (44–94)	169 (133–206)
3	342	74 (55–93)	50 (31–69)	124 (95–154)
4	308	50 (37–63)	52 (28–76)	102 (70–134)
5	274	50 (33–68)	66 (36–95)	116 (78–154)
6	235	35 (21–49)	81 (45–118)	116 (72–160)
7	197	38 (19–56)	80 (44–117)	118 (70–166)
8	162	40 (18–63)	81 (36–127)	122 (64–179)
9	136	36 (13–58)	79 (28–130)	114 (53–175)
10	113	43 (12–74)	73 (22–125)	117 (51–182)
11	94	43 (7–80)	31 (6–57)	75 (30–119)
12	75	30 (−2–61)	26 (−5–58)	56 (11–101)
13	48	15 (3–27)	26 (−14–65)	41 (−6–88)
14	24	23 (2–44)	0 (0–0)	23 (2–44)

Abbreviation: TSEK, Thousand Swedish Krona.

### 3.3 Sub‐Group Analyses

Among children born at 23 weeks, non‐medical costs peaked in Year 1 and declined thereafter, whereas children born at 21–22 weeks had more sustained non‐medical costs into later childhood: Figure S1. The distribution of costs between boys and girls showed a broadly similar pattern, with slightly higher non‐medical costs in boys during later years, though the subgroup sizes were small and not powered for statistical comparisons. The mode of delivery had minimal influence on long‐term cost trajectories: Figure S1. The total cost by age and subgroups is seen in Table S2.

**FIGURE 4:**
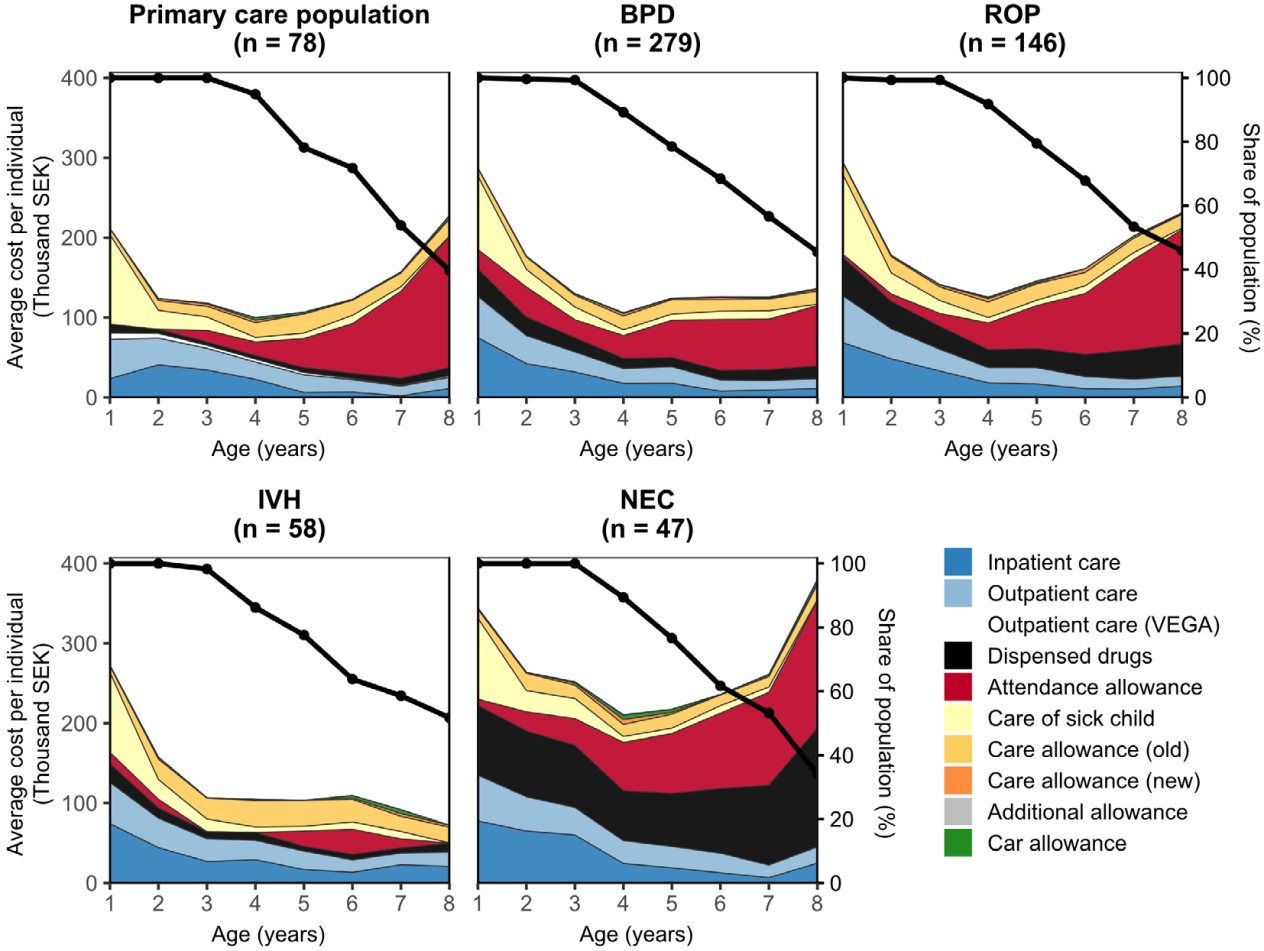
Mean cost (thousand SEK, Swedish Krona left y‐axis) by age (years) after infancy, among children born < 24 weeks GA top left Primary care population and subgroups included children with top middle bronchopulmonary dysplasia (BPD, *n* = 279/328), top right treated retinopathy of prematurity (ROP, *n* = 146/344), bottom left intraventricular haemorrhage grade 3–4 (IVH, *n* = 60/344), and bottom middle necrotising enterocolitis (NEC, *n* = 47/344). The black dotted line shows the proportion of the total number of infants per year (right y‐axis).

Figure 4 further illustrates the distribution of costs in the populations with specific neonatal morbidities, bronchopulmonary dysplasia, retinopathy of prematurity requiring treatment and severe intraventricular haemorrhage grade 3–4 and high overall societal costs, particularly due to increased support needs during later childhood.

## 4 Discussion

This nationwide population‐based study showed that births before 24 weeks of gestation were associated with substantial and sustained societal costs throughout childhood. Most medical costs occurred during the first year of life and were primarily driven by the need for neonatal intensive care. Non‐medical costs increased over time, particularly parental leave compensation and disability‐related allowances, and became a major component from preschool age. Neonatal morbidities appeared to be important indicators of long‐term cost patterns.

Our findings were supported by data from the United States showing that severe neonatal morbidities were associated with prolonged hospitalisation and elevated healthcare costs in preterm infants. A large multicentre cohort study by Lai and Lorch demonstrated that bronchopulmonary dysplasia, severe intraventricular haemorrhage and necrotising enterocolitis were major drivers of excess inpatient expenditure during the initial hospitalisation period [9]. Although their analysis was limited to the neonatal period and excluded non‐medical expenditure, the findings were consistent with our results in highlighting the disproportionate economic impact of specific neonatal complications. By extending the analysis beyond hospital discharge and including social insurance transfer costs, our study provided a more comprehensive picture of the long‐term economic impact associated with early‐life morbidity for both the health system and social insurance.

Although healthcare systems differ between countries, findings from Sweden's comprehensive welfare system can help indicate the types of long‐term support needs that may arise among children born extremely preterm and their families. These needs include services to adapt home or school environments and social insurance support that allows parents to remain at home to care for their children.

Our findings also aligned with Nordic register‐based evidence. The Finnish PERFECT project reported substantial early healthcare costs among very preterm children [11]. Our analysis had a longer follow‐up period and included a broader societal perspective by incorporating non‐medical transfer costs.

Other international studies reported similar patterns. Lai and Lorch found that bronchopulmonary dysplasia and intraventricular haemorrhage were associated with prolonged hospital stays and high inpatient expenditure in neonatal populations [9]. Studies from Canada [5] and the United Kingdom [7] showed that costs extended into later childhood and included non‐medical domains. These included special education, which was unfortunately not captured in our study, and productivity losses among caregivers.

Our analysis was conducted over a longer time horizon than these studies and used high‐quality individual‐level data from a publicly funded healthcare system. The results also extended those reported by Razaz et al., who showed elevated healthcare utilisation among preterm children in Sweden, particularly among those with early complications [25]. While that study focused on service use, our results linked neonatal morbidity to cumulative economic impact and complemented existing risk stratification frameworks.

Our findings have a number of policy implications. For example, they underscore the need for early and integrated care planning for infants born at the threshold of viability. The shift from acute inpatient costs to long‐term social support suggested that post‐discharge planning should extend beyond standard medical follow‐up. Collaboration between sectors, including health care, education and social services is likely to be important to support families and optimise outcomes. Our data also provided economic justification for structured transitional care models and proactive child and family support [26, 27].

Persistent non‐medical costs indicated less visible societal costs that may not always be recognised in clinical settings. Integrating cost‐related outcomes into national quality registries could help identify families with high support needs and guide tailored interventions.

Our findings also reflected the clinical heterogeneity among children born at the threshold of viability. Swedish cohort studies reported gestational age‐specific differences in survival and selected neonatal morbidities between infants born at 22 and 23 weeks of gestation. These studies also reported sex differences in specific outcomes, such as bronchopulmonary dysplasia among infants born at 23 weeks [10]. Such differences justified analysing subgroups separately because care trajectories, complication rates and long‐term support needs were not uniform.

Patterns of higher resource use in some subgroups were consistent with previously reported differences in neonatal morbidity. These patterns may inform future strategies for tailored follow‐up and long‐term support.

The heterogeneity and long‐term conditions experienced by these children and their families highlighted the need for structured support systems throughout childhood. These systems should address both the needs of the child and the broader needs of families affected by extreme prematurity (27,28). Future research should examine how child‐centred, person‐centred and family‐centred approaches could improve outcomes and reduce long‐term cost trajectories, particularly among high‐risk groups.

### 4.1 Strengths and Limitations

A major strength of this study was the use of high‐resolution population‐based data from multiple national registers with complete coverage. These data enabled reliable estimation of both medical and non‐medical costs over a long follow‐up period.

However, several limitations should be noted. Diagnosis‐related group cost estimates provide national comparability but do not represent true individual‐level costs. Instead, they represent standardised reimbursement tariffs applied to groups of healthcare encounters expected to require similar resources. These estimates may therefore not fully capture variation in resource use in cases of unusually complex or prolonged care.

Outpatient primary care was not included in diagnosis‐related group data during the study period and regional coverage varied slightly. Social insurance transfers are influenced by parental income, eligibility criteria and regional assessment practices, which may introduce socioeconomic variation. However, this limitation is less problematic in a descriptive analysis such as ours.

Children born abroad or those who emigrated during follow‐up were not captured in Swedish registers, which may have resulted in a slight underestimation of national costs. Children who died during childbirth or early infancy were also not included in the cost calculations. In addition, productivity loss, informal care, special education and out‐of‐pocket expenses were not included in the analysis.

Another limitation is that no statistical hypothesis testing was performed. Subgroup comparisons should therefore be interpreted as descriptive patterns rather than statistically confirmed differences.

## 5 Conclusion

Birth before 24 weeks of gestation was associated with substantial and persistent societal costs. These costs were initially driven by intensive neonatal care and later reflected long‐term support needs for children and families.

The findings highlight the importance of early planning for coordinated long‐term follow‐up and family support. They also underline the need for interdisciplinary and family‐centred approaches throughout childhood.

These results do not imply changes in treatment decisions for conditions such as necrotising enterocolitis or retinopathy of prematurity. Instead, they emphasise the need for structured long‐term support systems for children born extremely preterm and their families.

## Author Contributions

**Chatarina Löfqvist:** conceptualisation; methodology; project administration; writing – original draft; writing – review and editing; supervision. **Boubou Hallberg:** conceptualisation; investigation; methodology; writing – review and editing. **Ulrika Sjöbom:** visualisation; formal analysis, software, writing – review and editing. **David Ley:** methodology; writing – review and editing; validation. **Liv Vallin:** investigation; writing – review and editing. **Eva Morsing:** conceptualisation; resources; writing – review and editing. **Karin Sävman:** conceptualisation; methodology; writing – review and editing. **Ann Hellström:** conceptualization; funding acquisition; resources; supervision; writing – review and editing. **Hanna Gyllensten:** conceptualisation; methodology; formal analysis; data curation; software; visualisation; writing – review and editing; project administration.

## Funding

This work was supported by Vetenskapsrådet, 2020–01092. Knut och Alice Wallenbergs Stiftelse.

## Conflicts of Interest

The authors declare no conflicts of interest.

## Supporting information


**Figure S1:** Mean cost (thousands of SEK; left y‐axis) by age (years) after infancy, among children born < 24 weeks GA subgroups included (top panel) gender, (middle panel) gestational age group and (bottom panel) mode of delivery. The black line shows the proportion of the total number of infants per year (right y‐axis).
**Table S1:** Counts of healthcare contacts and social insurance benefits by age are available as an Excel file.
**Table S2:** Mean cost by age and subgroups. TSEK = Thousand Swedish Krona.

## Data Availability

The data that support the findings of this study are available from the corresponding author upon reasonable request.
