# Supplementary material for: Long‐Term Societal Costs After Births Before 24 Weeks of Gestation in Sweden
Source: Acta Paediatr. 2026 Mar 31;115(8):1638–45. doi: 10.1111/apa.70527 (PMC13371820; doi:10.1111/apa.70527)
Supplement: Supplementary file 1 — Figure S1: Mean cost (thousands of SEK; left y‐axis) by age (years) after infancy, among children born < 24 weeks GA subgroups included (top panel) gender, (middle panel) gestational age group and (bottom panel) mode of delivery. The black line shows the proportion of the total number of infants per year (right y‐axis). Table S1: Counts of healthcare contacts and social insurance benefits by age are available as an Excel file. Table S2: Mean cost by age and subgroups. TSEK = Thousand Swedish Krona. [file APA-115-1638-s001.zip › Supplement HE resubmitt 2 20260317.docx]

# Supplement

# Long-term Societal Costs after Births Before 24 Weeks of Gestation in Sweden

Chatarina Löfqvist^1,2,3^, Boubou Hallberg^4^, Ulrika Sjöbom^1,3^, David Ley^5^, Liv Vallin^6,7^, Eva Morsing^5^, Karin Sävman^6,7^, Ann Hellström^3,8^, Hanna Gyllensten^1,2^

Affiliations:

1 Institute of Health and Care Sciences, Sahlgrenska Academy, University of Gothenburg, Gothenburg, Sweden

2 University of Gothenburg Centre for Person-centred Care - GPCC, University of Gothenburg, Gothenburg, Sweden

2 Department of Clinical Neuroscience, Institute of Neuroscience and Physiology, Sahlgrenska Academy, University of Gothenburg, Gothenburg, Sweden

4 Sahlgrenska University Hospital, Gothenburg, Sweden

5 Department of Pediatrics, Institute of Clinical Sciences, Skåne University Hospital Lund, Lund, Sweden

6 Department of Neonatology, The Queen Silvia Children’s Hospital, Sahlgrenska University Hospital, Gothenburg, Sweden

7 Department of Pediatrics, Institute of Clinical Sciences, Sahlgrenska Academy, University of Gothenburg, Gothenburg, Sweden

8 Department of Ophthalmology, Sahlgrenska University Hospital, Gothenburg, Sweden

**Figures**

**Figure S1**. Mean cost (thousands of SEK; left y-axis) by age (years) after infancy, among children born <24 weeks GA subgroups included (top panel) gender, (middle panel) gestational age group and (bottom panel) mode of delivery. The black line shows the proportion of the total number of infants per year (right y-axis).


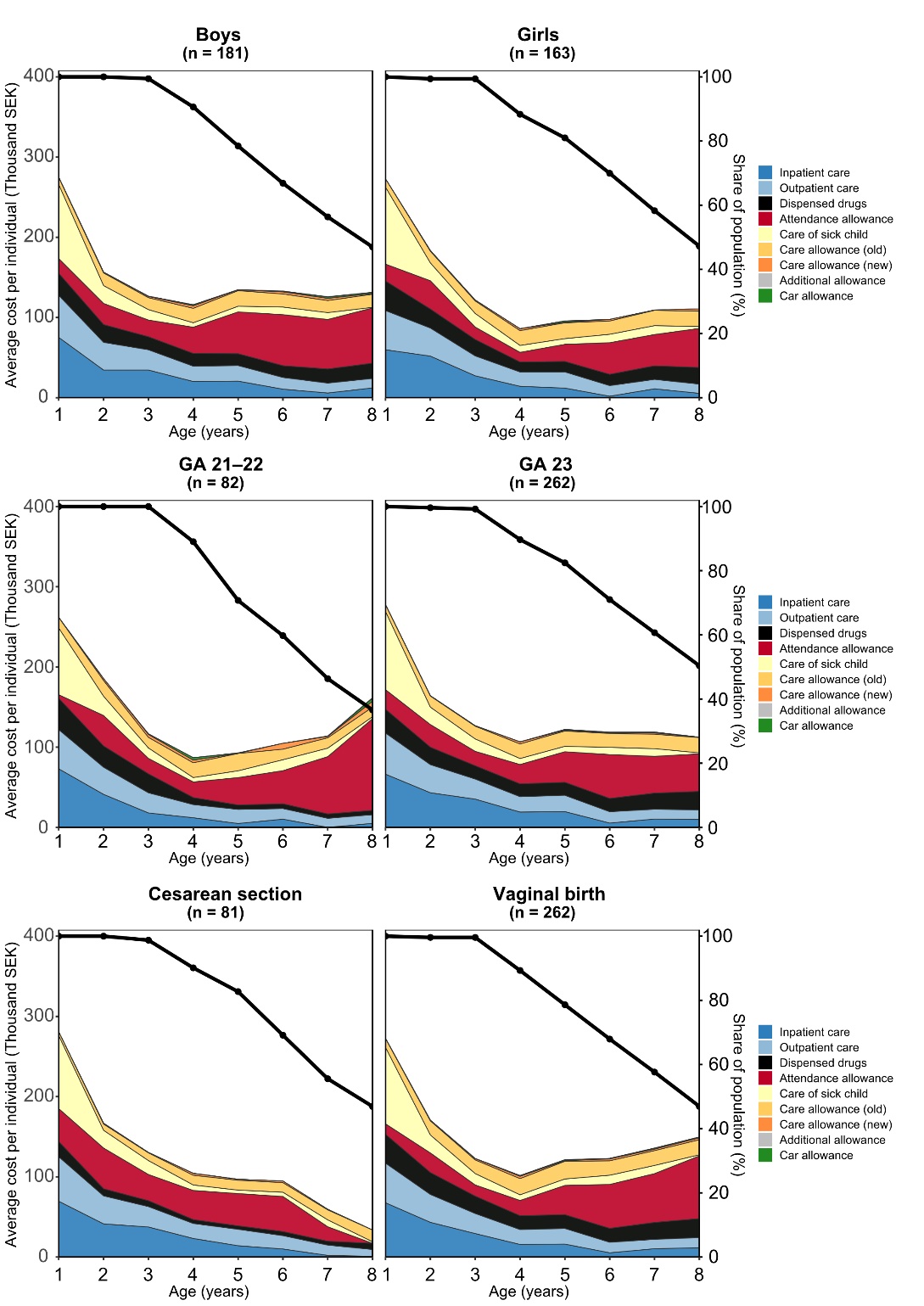


**Tables**

**Table S1:** Counts of healthcare contacts and social insurance benefits by age are available as an Excel file.

**Table S2:** Mean cost by age and subgroups. TSEK= Thousand Swedish Krona

|  | | Mean (95% CI) TSEK | | | | | |
| --- | --- | --- | --- | --- | --- | --- | --- |
| Year  of  Age | **Girl** | | **Boy** | **w21-22** | **w23** | **No caesarean section** | **Caesarean section** |
| 0 | 1624  (1495-1752) n=163 | | 1724  (1585-1862) n=181 | 1707  (1496-1917) n=82 | 1667  (1560-1773) n=262 | 1633  (1524-1742) n=262 | 1825  (1630-2020) n=81 |
| 1 | 273  (224-322) n=163 | | 275  (227-323) n=181 | 262  (209-315) n=82 | 278  (236-320) n=262 | 273  (236-309) n=262 | 281  (195-367) n=81 |
| 2 | 183  (124-243) n=162 | | 157  (112-201) n=181 | 186  (122-249) n=82 | 164  (121-208) n=261 | 171  (130-211) n=261 | 167  (84-250) n=81 |
| 3 | 122  (83-161) n=162 | | 127  (83-170) n=180 | 117  (64-169) n=82 | 127  (92-162) n=260 | 123  (92-154) n=261 | 130  (55-206) n=80 |
| 4 | 86  (53-119) n=144 | | 116  (63-169) n=164 | 87  (38-135) n=73 | 107  (67-146) n=235 | 102  (68-135) n=234 | 105  (20-189) n=73 |
| 5 | 96  (52-139) n=132 | | 135  (73-197) n=142 | 93  (36-150) n=58 | 122  (76-168) n=216 | 121  (78-165) n=206 | 97  (15-179) n=67 |
| 6 | 98  (45-150) n=114 | | 133  (63-202) n=121 | 105  (30-180) n=49 | 119  (67-170) n=186 | 123  (73-173) n=178 | 95  (4-186)  n=56 |
| 7 | 109  (40-178) n=95 | | 126  (58-194) n=102 | 114  (20-208) n=38 | 119  (63-174) n=159 | 136  (74-198) n=151 | 59  (20-99)  n=45 |
| 8 | 111  (34-188) n=77 | | 131  (46-216) n=85 | 162  (27-296) n=30 | 112  (49-176) n=132 | 150  (75-225) n=123 | 34  (23-45)  n=38 |
| 9 | 110  (22-198) n=67 | | 119  (34-204) n=69 | 125  (-10-260) n=26 | 112  (43-180) n=110 | 138  (60-215) n=106 | 32  (17-47)  n=30 |
| 10 | 128  (29-226) n=54 | | 106  (19-194) n=59 | 91  (-27-209) n=20 | 122  (46-198) n=93 | 141  (56-227) n=86 | 37  (16-59)  n=27 |
| 11 | 70  (13-126) n=46 | | 79  (10-148) n=48 | 117  (-54-287) n=14 | 67  (24-110) n=80 | 88  (31-146) n=72 | 30  (11-49)  n=22 |
| 12 | 58  (-5-122) n=37 | | 53  (-12-118) n=38 | 131  (-93-354) n=11 | 43  (6-80) n=64 | 67  (8-125) n=58 | 20  (6-33)  n=17 |
| 13 | 27  (3-51) n=22 | | 52  (-33-137) n=26 | 166  (-150-482) n=7 | 19  (6-32) n=41 | 49  (-11-110) n=37 | 11  (3-20)  n=11 |
| 14 | 28  (-11-67) n=12 | | 18  (1-34) n=12 | 43  (-36-121) n=2 | 21  (-1-43) n=22 | 14  (3-25) n=20 | 67  (-50-183) n=4 |
